# Body fluids from the rat exposed to chlorpyrifos induce cytotoxicity against the corresponding tissue−derived cells *in vitro*

**DOI:** 10.1186/s40360-021-00531-9

**Published:** 2021-10-20

**Authors:** Yu-Jie Liang, Ding-Xin Long, Ming-Yuan Xu, Hui-Ping Wang, Ying-Jian Sun, Yi-Jun Wu

**Affiliations:** 1 Department of Veterinary Medicine and Animal Science, Beijing University of Agriculture, 102206 Beijing, PR China; 2 Laboratory of Molecular Toxicology, Institute of Zoology, Chinese Academy of Sciences, 100101 Beijing, PR China; 3 School of Public Health, University of South China, 421001 Hengyang, P. R. China

**Keywords:** Pesticide, Exposure, Assessment, Body fluid, Cytotoxicity, Tissue-derived cell

## Abstract

**Background:**

This study aims to establish an *in vitro* monitoring approach to evaluate the pesticide exposures. We studied the *in vitro* cytotoxicity of three different body fluids of rats to the respective corresponding tissue-derived cells.

**Methods:**

Wistar rats were orally administrated daily with three different doses of chlorpyrifos (1.30, 3.26, and 8.15 mg/kg body weight/day, which is equal to the doses of 1/125, 1/50, and 1/20 LD_50_, respectively) for consecutive 90 days. Blood samples as well as 24-hour urine and fecal samples were collected and processed. Then, urine, serum, and feces samples were used to treat the correspondent cell lines, i.e., T24 bladder cancer cells, Jurkat lymphocytes, and HT-29 colon cancer cells respectively, which derived from the correspondent tissues that could interact with the respective corresponding body fluids in organism. Cell viability was determined by using MTT or trypan blue staining.

**Results:**

The results showed that urine, serum, and feces extract of the rats exposed to chlorpyrifos displayed concentration- and time-dependent cytotoxicity to the cell lines. Furthermore, we found that the cytotoxicity of body fluids from the exposed animals was mainly due to the presence of 3, 4, 5-trichloropyrindinol, the major toxic metabolite of chlorpyrifos.

**Conclusions:**

These findings indicated that urine, serum, and feces extraction, especially urine, combining with the corresponding tissue-derived cell lines as the *in vitro* cell models could be used to evaluate the animal exposure to pesticides even at the low dose with no apparent toxicological signs in the animals. Thus, this *in vitro* approach could be served as complementary methodology to the existing toolbox of biological monitoring of long-term and low-dose exposure to environmental pesticide residues in practice.

## Introduction

Due to the widespread use of pesticides worldwide, people are subject to environmental exposure to pesticide residues at some levels almost inevitably [1, 2]. Thus, assessing the environmental exposure to humans is of great importance to the risk assessment [3]. The biological monitoring of exposure is a powerful tool for assessing environmental exposures to toxicants, which either determines the internal dose of a chemical by measuring the concentration of a chemical and its metabolites in given biologic matrices or measures the early biological effects of pesticides such as inhibition of acetylcholinesterase [4–6]. Urine and blood are two major matrices that are used to assess exposure to chemicals [7, 8]. However, the measurement of organic chemicals requires expensive instrumentations and highly trained analysts, which makes the measurement very costly, ranging from $100 to $1500 per sample depending on the sensitivity of the measurement [4]. And the dose-effect relationships for the measurement of the biological effectors such as the inhibition of acetylcholinesterase by pesticides are not fully established [5]. Thus, alternative approaches are needed for assessing the environmental exposure.

Various disease states could lead to drastic change in body fluids such as urine and serum [9]. Thus body fluids have been used in the diagnosis of various diseases; for example, a study showed that urine samples from patients with interstitial cystitis, a chronic bladder disease, could cause cytotoxicity to a bladder epithelial cell line *in vitro* [10, 11]. Thus, the measurement of the antiproliferative activity of urine in the bladder-derived cell lines may be a useful noninvasive method for the diagnosis of bladder diseases [12]. Another example is that serum from patients with multiple sclerosis could cause demyelination in the cultured rat nerve tissues and induce cytotoxicity to the cultured rat oligodendrocytes [13].

However, the use of this indirect approach in exposure assessment to toxic chemicals has not been thoroughly explored. In this study, we used rats exposed chronically to low dose chlorpyrifos, a commonly used organophosphorus pesticide (OP), as the model for human and other mammalians exposure to environmental OPs. We sought to establish the indirect biological monitoring approach for environmental exposure assessment by measuring the cytotoxicity of different biological matrices from the chlorpyrifos-exposed rats in the following *in vitro* cell models. Bladder cells T24 were treated with correspondent urine samples. Colon cancer cells HT-29 were treated with correspondent feces extracts. Jurkat lymphocyte cells were treated with correspondent serum samples. MTT staining and trypan blue staining were used to measure cell viability. The cell model with the highest sensitivity and specificity may be used as to assess the environmental exposure and to study the mechanisms of toxicity of pesticides as well as other environmental pollutants.

## Materials and methods

### Materials

Chlorpyrifos (purity > 95 %) was obtained from Shuangma Chemical Co. Ltd. (Jiangsu, China). RPMI-1640, dimethyl sulfoxide (DMSO), and 3-(4,5-dimethylthiazol-2-yl)-2,5-diphenyltetrazolium bromide (MTT) were obtained from Sigma Aldrich Co. (St Louis, MO, USA). Dulbecco’s modified Eagle’s medium (DMEM) was obtained from Life Technologies (Carlsbad, CA, USA). Heat-inactivated fetal bovine serum (FBS) was obtained from Chuanye Biotechnology, Ltd (Tianjin, China). Bladder cancer cell line T24, colon cancer cell line HT-29 and lymphocyte cell line Jurkat were purchased from Cell Center of Chinese Academy of Medical Sciences (Beijing, China).

### Animals and treatment

**Fig. 1:**
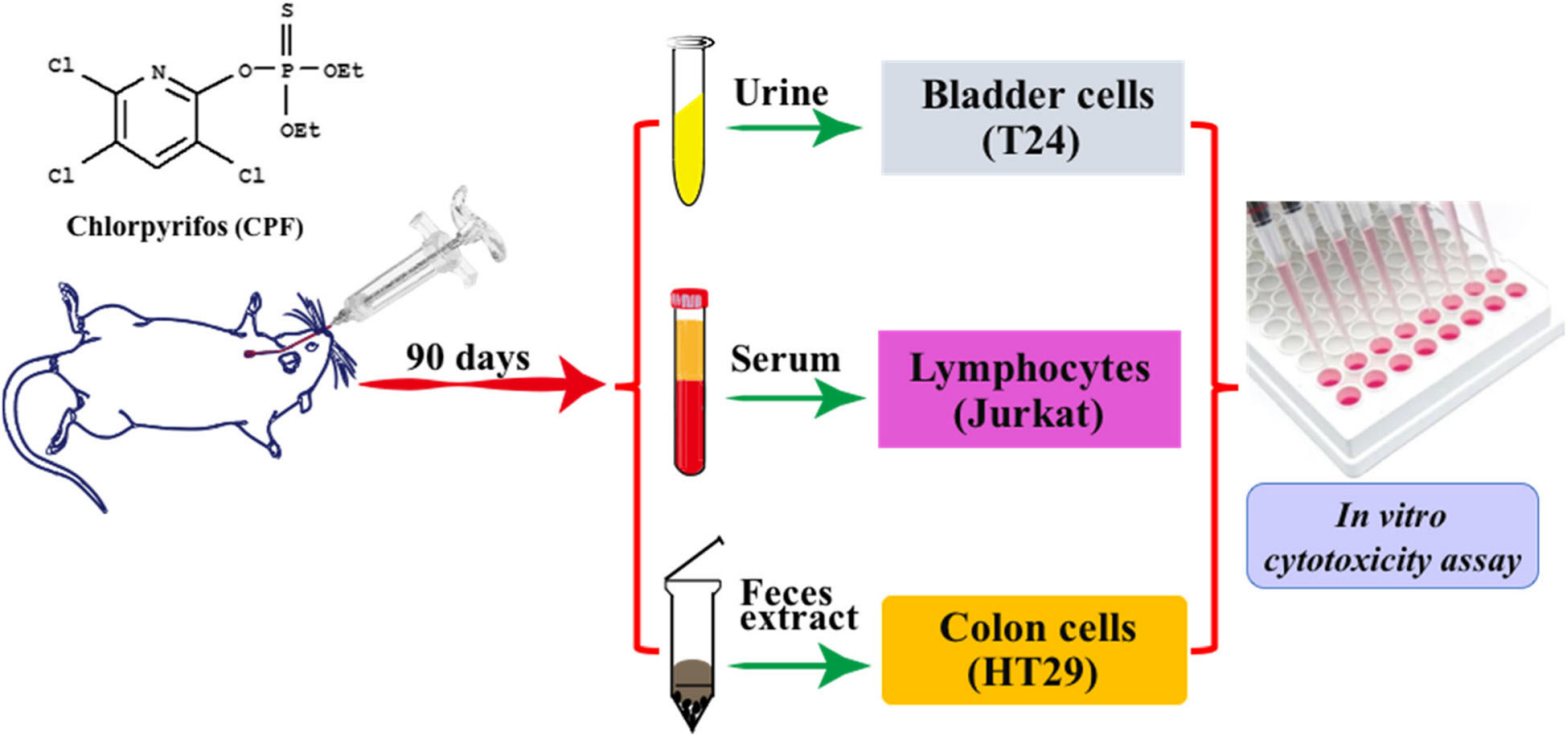
Schematic illustration of the experiments with *in vitro* cytotoxicity of body fluids of rats toward the respective corresponding tissue-derived cells to reveal adverse effects of low-dose long-term exposure of chlorpyrifos

Forty 6-8-week old male Wistar rats with body weight of 150–220 g were obtained from Laboratory Animal Technology Company (Beijing, China). The animals were maintained at dark/light cycle of 12 h, 22 ± 2 °C and 55 ± 10 % humidity. The rats were kept individually with free access to feed and water. Based on data from previous studies that the acute oral half-lethal doses (LD_50_) of chlorpyrifos were 163 mg/kg for male rat [14], we chose the doses of 1/125, 1/50, and 1/20 LD_50_ of chlorpyrifos as low dose (1.30 mg/kg body weight), medium dose (3.26 mg/kg body weight), and high dose (8.15 mg/kg) for the pesticide treatment groups in this study. The low dose of chlorpyrifos (1.30 mg/kg body weight) is close to the level of possible occupational exposure [15]. After 7 days of acclimatization, the animals were divided into three chlorpyrifos treatment groups (low dose: 1.30 mg/kg, medium dose: 3.26 mg/kg, and high dose: 8.15 mg/kg) and one vehicle control group with 10 rats in each group. The pesticide was dissolved in corn oil (0.5 ml/kg body weight) before being orally administered daily. The control group received an equivalent volume of corn oil daily. All of the animals were treated for consecutive 90 days to mimic the chronic exposure to pesticides in humans. At the end of the experiment, the samples of urine, feces, and blood were collected once from each of the animals (Fig. 1).

All animal procedures were performed in accordance with current China legislation and approved by the Animal and Medical Ethics Committee, which is affiliated to the Institute of Zoology, Chinese Academy of Sciences.

### Preparation of body fluids samples

Ten milliliters (10 mL) of the collected 24-hour urine samples were processed with protein precipitation; the supernatant was lyophilized and suspended in 1 mL of phosphate buffer solution (PBS). The solution was sterilized by filtration with 0.22-µm filters, and then diluted ten times by complete cell culture medium to make a stock solution, which was further diluted to treat cells to determine the dose-response effect of urine extract on cell viability.

Feces collected from animals were suspended in 0.1 M cold PBS (1:5, w/v) and then incubated with PBS buffer overnight with stirring. The solution was centrifuged at 3, 000 × g for 30 min at 4 °C and then the supernatant was centrifuged at 15, 000 × g for 30 min at 4 °C. The supernatant was sterilized by filtration with 0.22 μm filters and diluted ten times with cell culture medium, which was further diluted to treat cells to determine the dose-response effect of feces extract on cell viability.

Serum collected from the animals was heated at 56 °C for 30 min to inactivate complement proteins and potential mycoplasma, and then the serum was diluted ten times in complete cell culture medium containing 10 % FBS to treat cells to determine the dose-response effect of the serum on cell viability.

### Cell culture and treatment

The cells were cultured with DMEM (for T24 and HT-29 cell lines) or RPMI-1640 (for Jurkat cell line) plus 10 % FBS complemented with 100 U/mL penicillin and 100 µg/mL streptomycin. The cells were maintained at 37 °C in a humidified atmosphere containing 5 % CO_2_.

### MTT staining

MTT staining was carried out according to the reference [16]. Briefly, T24 and HT-29 cells were seeded in 96-well plates with 1 × 10^4^ cells/well. After 24 h, the urine stock solutions were added to T24 cells with different dilutions. Feces extracts were added to HT-29 cells with different dilutions. The corresponding urine and feces extracts from the vehicle-treated rats were also used to treat the cells, which served as vehicle controls. Culture medium was used as the negative control. After cells were treated for different time periods, 20 µl of MTT (5 mg/ml) was added to each well. Four hours later, the cell culture medium was discarded and 150 µl of DMSO was added to each well. OD_570_ values were measured by using Bio-Rad Benchmark microplate reader. Each treatment was performed in triplicate and each experiment was repeated at least three times.

To eliminate possible non-specific interference from the samples, we determined the absorbance of the culture medium sparked with the prepared urine stock solution and feces extract samples under the cell-free condition (all other operations are the same as the MTT assay except for in the absence of cells). The result showed that no difference of the OD_570_ values was found between the culture medium group (blank) and the spiked medium group.

### Trypan blue staining

Jurkat cells were seeded in 12-well plate with 1 × 10^5^ cells/well. After 12 h, the cells were treated with 300 µl of the 10-fold diluted serum from the rats treated with 1.30 mg/kg, 3.26 mg/kg, or 8.15 mg/kg chlorpyrifos, respectively. After cultured for different time periods, cells were stained by trypan blue and the live cell number in each well was counted. Serum samples from the corn oil-treated rats were used as the vehicle control and 10 % FBS in regular cell culture medium was used as the negative control. Each treatment was performed in triplicate and each experiment was repeated at least three times.

### Determination of chlorpyrifos and TCP levels in the serum and urine

The assay of chlorpyrifos and its metabolite 3, 4, 5-trichloropyrindinol (TCP) was carried out according to the method of the previous study [17]. Briefly, 200 µl of the serum or urine were added to 600 µl of acetic ether and vortexed for 1 min. The solution was centrifuged at 1, 000 × g for 20 min and then the supernatants were collected, dried under nitrogen and then dissolved in 150 µl of methanol. The solution was centrifuged at 15, 000 × g for 20 min and then the supernatants were transferred into the vials. The samples were analyzed by the Agilent 1100 series HPLC system (Agilent Co., USA).

### Statistics

All data are presented as mean ± Standard Error of the Mean (SEM). Statistical differences between treated and control groups were evaluated by GraphPad Prism 8.0 (SanDiego, CA, USA). The one-way Analysis of Variance (ANOVA) test followed by the post hoc Dunnett’s test was performed to access significant differences among all the groups. The difference was considered significant if the p-value was less than 0.05.

## Results

### Symptoms and body weight changes of CPF-treated rats

There were no deaths of the rats during the course of the whole study. Rats treated with chlorpyrifos for 90 consecutive days did not demonstrate overt signs of toxicity probably due to the very low dose of chlorpyrifos used in this study. The rats in all treatment groups progressively gained weight over the 90-day period; however, there were no significant differences between body weights of chlorpyrifos-treated rats and that of vehicle control rats (data not shown).

### The cytotoxicity of the urine prepared to T24 bladder cells

**Fig. 2:**
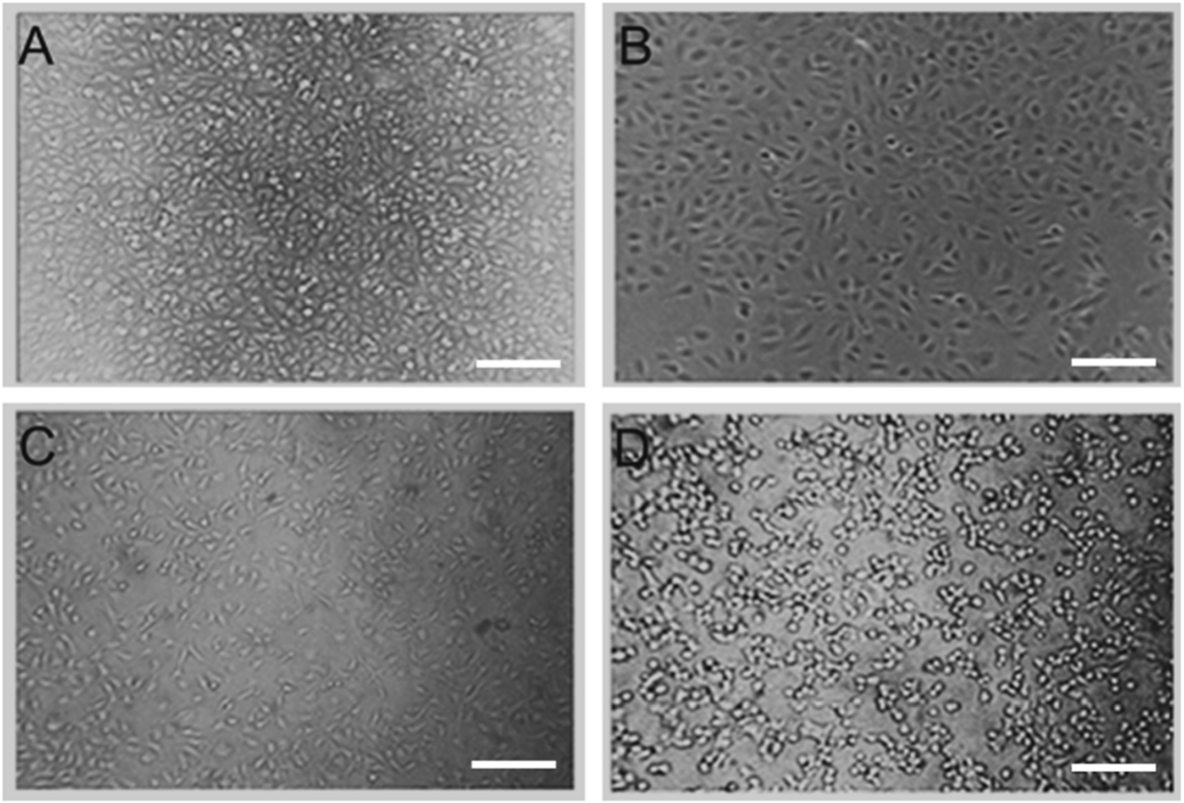
Morphological changes of T24 cells treated with the urine prepared from rats exposed to chlorpyrifos (CPF) at doses of 0 mg/kg/day (**A**), 1.30 mg/kg/day (**B**), 3.26 mg/kg/day (**C**), and 8.15 mg/kg/day (**D**). Scale bars = 200 μm

Bladder cancer cell line T24 cells were treated with the urine prepared from the rats exposed to chlorpyrifos. The cell shape became round and the cytosol was condensed (Fig. 2). The changes were more prominent in groups treated with urine samples from the rats exposed to high dose of chlorpyrifos (8.15 mg/kg/day) (Fig. 2).

**Fig. 3:**
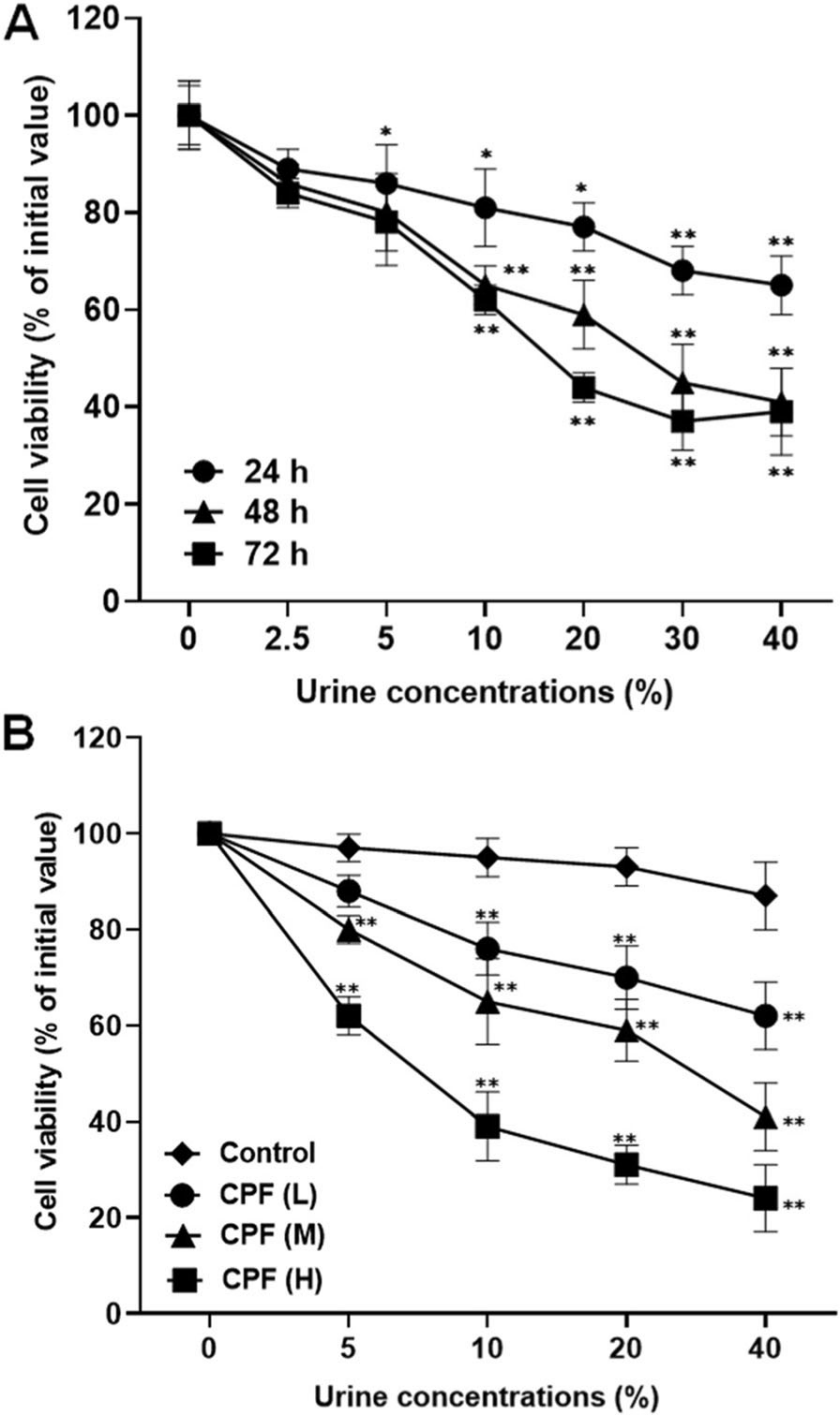
Cytotoxicity of the urine from rats exposed to chlorpyrifos (CPF). Cell viability was determined using the MTT assay after the T24 cells were treated for different time by the urine prepared from the rats exposed to medium dose of CPF (3.26 mg/kg/day) (**A**), or treated for 48 h with the urine prepared from rats exposed to CPF at doses of 1.30 mg/kg/day (L), 3.26 mg/kg/day (M), and 8.15 mg/kg/day (H) respectively (**B**). ^*^P < 0.05 and ^**^P < 0.01, compared with the control

The cell viability of T24 cells decreased after treated with urine samples from the rats exposed to the medium dose of chlorpyrifos (3.26 mg/kg/day) (Fig. 3 A). The cytotoxicity increased in a concentration- and cell exposure time-dependent manner. Treatment for 72 h had a similar effect to the treatment for 48 h. Thus the following experiments used 48-hour treatment. The cytotoxicity was higher in cells treated with urine samples from the rats exposed to higher dose of chlorpyrifos (Fig. 3B).

### The cytotoxicity of the feces extracts to HT-29 colon cells

**Fig. 4:**
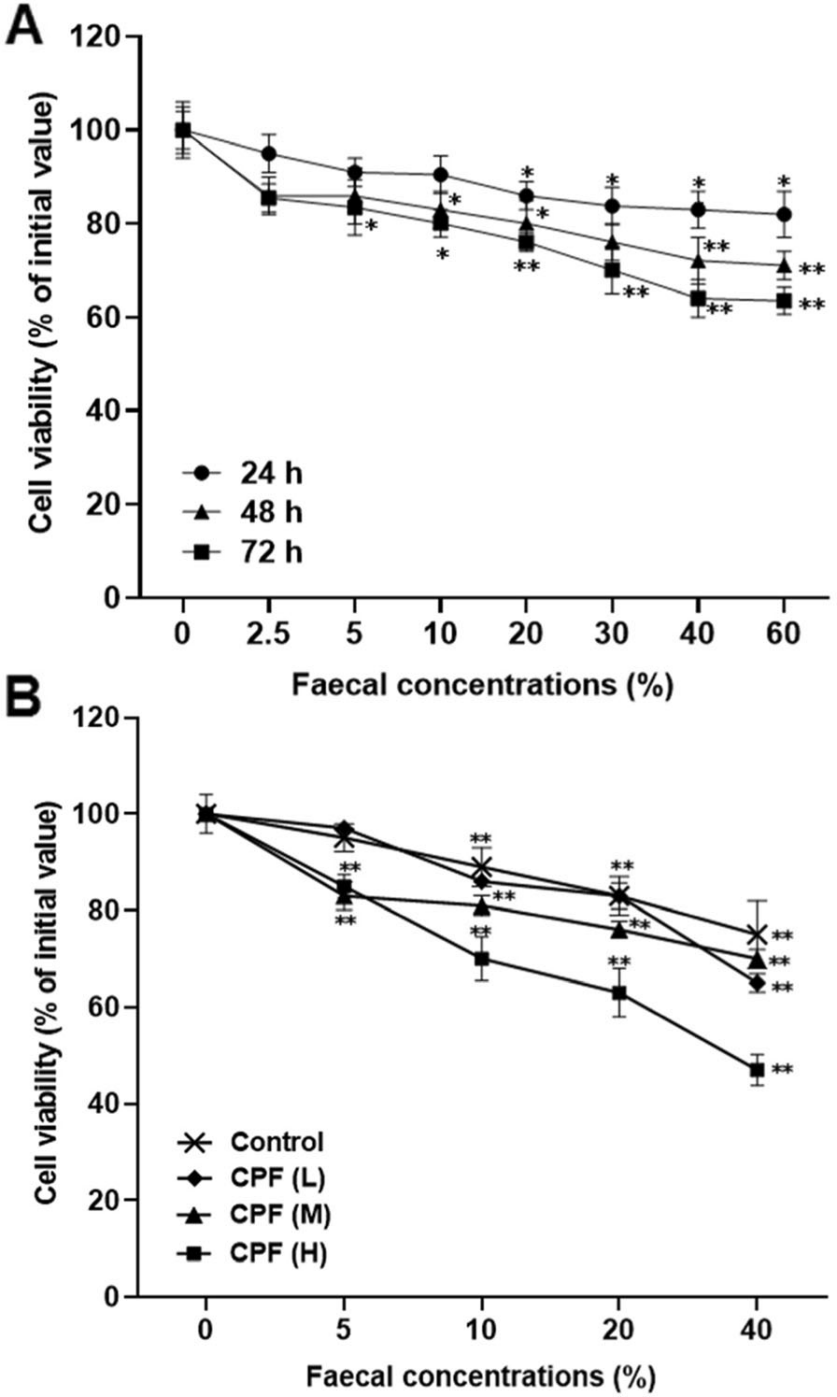
Cytotoxicity of the feces extracts from rats exposed to chlorpyrifos (CPF). Cell viability was determined using the MTT assay after the HT-29 cells were treated by the feces extracts from the rats exposed to medium dose of CPF (3.26 mg/kg/day) for different time (**A**) or treated for 48 h with the feces extracts from rats exposed to CPF at doses of 1.30 mg/kg/day (L), 3.26 mg/kg/day (M), and 8.15 mg/kg/day (H), respectively (**B**). ^*^P < 0.05 and ^**^P < 0.01, compared with the control

The cell viability of colon cancer cell line HT-29 cells decreased after treated with feces extracts from the rats exposed to medium dose of chlorpyrifos (3.26 mg/kg/day) for 48 h (Fig. 4 A). The cytotoxicity escalated as the fecal treatment concentration and cell exposure time increased. The cytotoxicity was higher in cells treated with feces extract from the rats exposed to higher dose of chlorpyrifos (Fig. 4B). The cytotoxicity of the fecal control extract also increased significantly but less so than the chlorpyrifos exposure treatment samples (Fig. 4B).

### The cytotoxicity of the serum prepared to lymphocyte cells

**Fig. 5:**
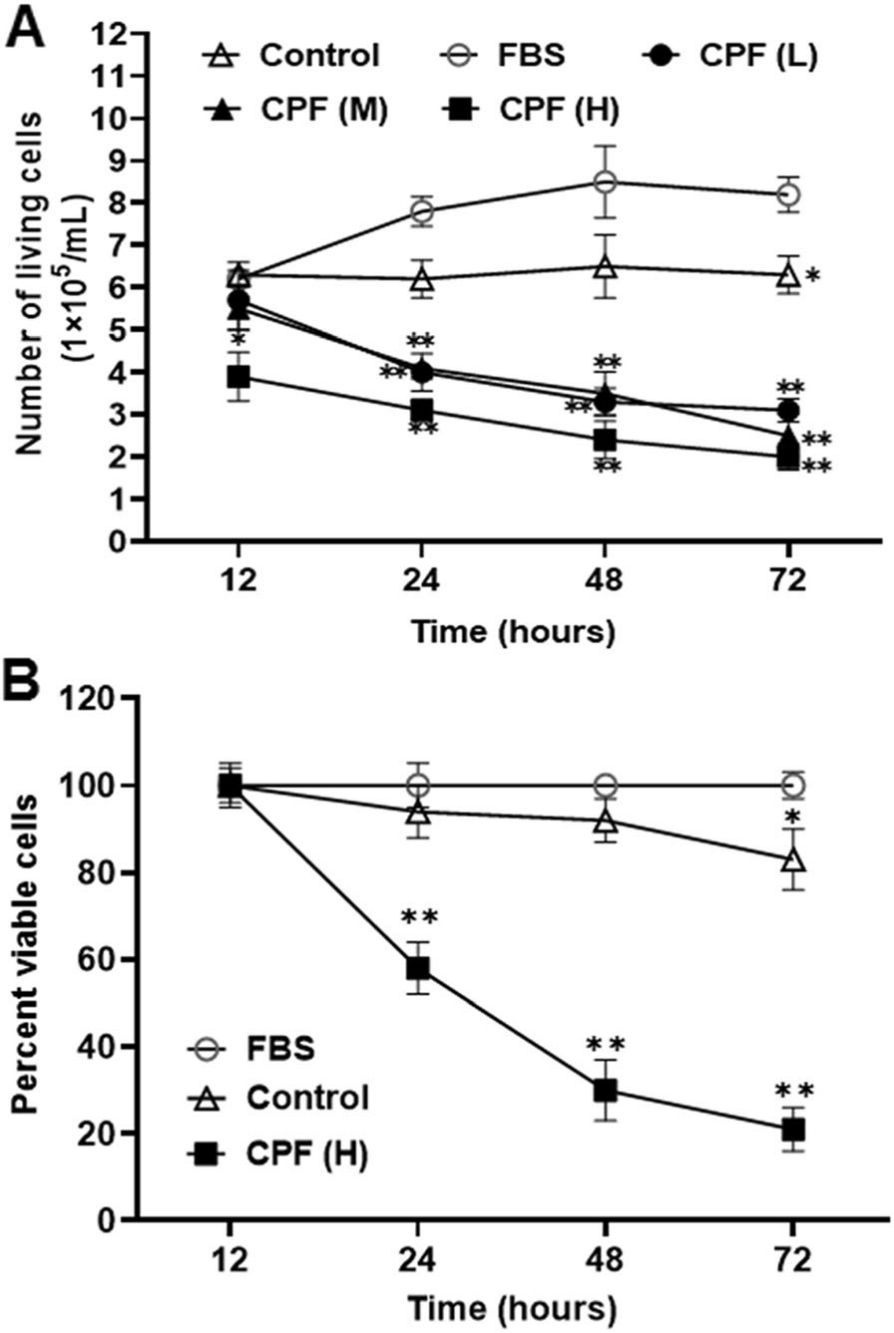
Cytotoxicity of the serum from rats exposed to chlorpyrifos (CPF). (**A**) Live cell number of Jurkat cells were determined after treated with serum prepared from the rats exposed to CPF at doses of 1.30 mg/kg/day (L), 3.26 mg/kg/day (M), and 8.15 mg/kg/day (H), respectively. (**B**) Viable cells were counted after Jurkat cells were treated with serum prepared from rats exposed to high dose of CPF (8.15 mg/kg/day). Abbreviations: FBS, fetal bovine serum. ^*^P < 0.05 and ^**^P < 0.01, compared with the control

The live cell number of Jurkat lymphocytes decreased after treated with the 10-fold diluted serum from the rats exposed to different doses of chlorpyrifos, as measured using the trypan blue assay (Fig. 5 A). The cytotoxicity increased in a chlorpyrifos concentration- and cell exposure time-dependent manner. After Jurkat cells were treated with serum samples from the rats exposed to high dose of chlorpyrifos for 24 h, the cell number significantly decreased 32.3 % and 44.6 % compared with that of the cells treated with serum samples from vehicle control rats or with 10 % FBS in regular cell culture medium (negative control cells), respectively (Fig. 5B). After treated for 72 h, the number of the cells significantly decreased 60.5 % and 79.7 % compared with that of the cells treated with serum samples from vehicle control rats or with 10 % FBS in regular cell culture medium (negative control cells), respectively (Fig. 5B).

### The levels of chlorpyrifos and its metabolite in the serum and urine

After 90-day chlorpyrifos exposure, the chlorpyrifos concentration in the serum detected was not obviously different among the three doses groups; however, the level of chlorpyrifos in the urine from the rats exposed to the high dose of chlorpyrifos (8.15 mg/kg/day) is higher than that in the other two doses groups. There is no obvious difference of the chlorpyrifos content between the serum and urine, while the levels of TCP, a chlorpyrifos metabolite, not only are much higher in urine than that in serum but also increased in both serum and urine as the administration dose increased (Table 1).

**Table 1:** The levels of chlorpyrifos and its metabolite TCP in the serum and urine of the rats exposed to different doses of chlorpyrifos for 90 days

Groups	CPF (µg/ml)		TCP (µg/ml)	
	Serum	Urine	Serum	Urine
Control	ND	ND	ND	ND
CPF-L	0.007 ± 0.001	0.007 ± 0.001	0.025 ± 0.007^*^	4.463 ± 0.808^*^
CPF-M	0.005 ± 0.000	0.008 ± 0.002	0.054 ± 0.008^*^	39.135 ± 8.521^*^
CPF-H	0.009 ± 0.002	0.021 ± 0.008^**^	0.084 ± 0.007^*^	58.853 ± 6.948^*^

Note: ^*^P < 0.05 and ^**^P < 0.01, compared with the control. Abbreviations: *ND* not detected, *CPF* chlorpyrifos, *TCP* trichloropyrindinol, *L* low dose, *M* medium dose, *H* high dose

## Discussion

The biological monitoring of organophosphorus pesticides exposure usually relies on the measurement of serum levels of the pesticide and its metabolite or acetylcholinesterase activity in the population [5]. The current study proposed a novel approach to monitor organophosphorus pesticide chlorpyrifos exposure in the population by measuring the cytotoxicity of body fluids to *in vitro* cultured cells (T24, HT-29, and Jurkat cells). The results showed that the cytotoxicity of the body fluids to the cultured cells reliably reflected the dose of chlorpyrifos administered to the tested animals.

After chemicals are absorbed by an organism into the circulation and distributed into organs, the chemicals are metabolized and excreted from the body. The residues of chlorpyrifos metabolite TCP are usually detected in the urine, blood, and other body fluids of human and other animals when exposed to chlorpyrifos [18–20]. And TCP has been used as a biomarker of chlorpyrifos exposure. However, TCP was found to be even more toxic than its parent compound chlorpyrifos [21–23]. Thus, the cytotoxicity of body fluids from the exposed animals was due to not only chlorpyrifos itself but also the presence of its metabolites, since the corresponding different levels of chlorpyrifos and much higher levels of the metabolite TCP were found in the urine and serum of the rats exposed to chlorpyrifos (Table 1). It suggests that TCP could be involved mainly in the cytotoxicity of chlorpyrifos exposure. Studies have clarified that TCP is the main factor in negative influence of chlorpyrifos [24]. Since bladder is the main organ for storing urine, the effect of TCP on the cells derived from bladder tissue is worthy of further study. Urine is best suited for non-invasive diagnosis and monitoring because it is easy to obtain and process. In addition, urine contains a variety of metabolic products [25]. Thus urine samples from vehicle-treated animals inhibited cell viability to some extent as well; however, the cytotoxicity of urine samples reflected the low-dose exposure of the pesticide.

We all know that traditional toxicological risk assessment focuses on the “direct” assay for the effect of chemical on cultured cells *in vitro* or laboratory animals *in vivo*. The traditional toxicology methods mainly rely on whole animal experiments to assess the impact of xenobiotics on human and ecological health. However, it is not easy to understand accurately the effect of a chemical on specific organs after being metabolized in the body especially at the existence of multiple metabolic products of the tested chemical in the body. Our results suggested that the cytotoxicity test analysis of body fluids from the animals exposed to pesticide could expand the application of the traditional toxicity tests *in vitro* and maximize the use of the *in vivo* animal experiment tests.

In this study, we used the cultured cell lines as cell models, which are corresponding to the organs of body, to do the toxicity test as a viable alternative. Overall, our results showed that urine, serum, and fecal extract can reflect the long-term exposure of pesticides even at environmental residue levels with no apparent toxicological signs. However, this cytotoxicity endpoint assay is not a reliable surrogate for quantitative exposure biomonitoring where a specific chemical is identified and quantified in a biological compartment. We expected that in the future studies, more advanced effective alternatives such as primary cells from the corresponding organs, 3D cell models, and tissue slices would be used for more accurately evaluating the changes of body environment to identify the toxicities of the exposed chemicals.

## Conclusions

This study proposes that the cytotoxicity measurement of the cells, which are from three organ-derived cell lines, incubated with the corresponding body fluids could be used to monitor the exposure and toxicity of the residues of pesticides in the body. This simple assay *in vitro* approach could be served as a complementary methodology to identify the toxicities of the long-term and low-dose exposure of pesticides or other environmental pollutants.

## Data Availability

All data generated or analyzed during this study are included in this published article.
